# In vivo cardiopulmonary impact of skeletal M_3_Dq DREADD expression: a pilot study

**DOI:** 10.1007/s00360-021-01387-5

**Published:** 2021-07-16

**Authors:** Sandra G. Vincent, John T. Fisher

**Affiliations:** Department of Biomedical and Molecular Sciences and Division of Respirology, Department of Medicine, Queen’s University, Kingston, ON K7L 3N6 Canada

**Keywords:** Muscarinic acetylcholine receptor, DREADD, Thermoregulation, Heart rate, In vivo cardiopulmonary physiology, Mouse

## Abstract

The muscarinic M_3_ receptor (M_3_R) is implicated in cardiopulmonary control and many other peripheral physiologic functions. Previous observations report mortality in mice expressing a Gq-linked designer G-protein coupled receptor (Dq) selectively in striated muscle, while M_3_Dq DREADD (Designer Receptor Exclusively Activated by Designer Drug), selectively expressed in skeletal muscle (SKM) impacts glucose metabolism. We investigated whether activation of SKM M_3_Dq impacts cardiopulmonary function. Heart rate (HR), body temperature (Tb) and locomotor activity (ACT) were measured in 4 conscious, chronically instrumented M_3_Dq DREADD mice and 4 wildtype controls. Circadian values of HR, BT and ACT were not different between genotypes (*p* > 0.05). Activation of the M_3_Dq DREADD by clozapine N-oxide (CNO; 0.1 mg/kg) resulted in: a significant drop in heart rate, 2 h after injection, compared with a time-matched baseline control period from the same animals (460 ± 28 vs. 532 ± 6, *p* < 0.05), significantly lower ACT compared to the baseline control (*p* < 0.05) and reduced pulmonary minute ventilation compared to pre-CNO control (*p* < 0.05). M_3_Dq DREADD activation did not cause bronchoconstriction (separate protocol), however, there was a concomitant reduction in HR, Tb and ventilation, accompanied by cardiac arrhythmias. We speculate that reductions in Tb, HR and ventilation reflect a mechanistic link between SKM Gq signaling and the metabolic responses associated with the initiation of torpor. Supported by the Canadian Institutes of Health Research (CIHR MOP-81211).

## Introduction

In small mammalian species, including the mouse, the coordinated control of metabolism, minute ventilation and body temperature is an important adaptive response to environmental stimuli (Frappell et al. 1992; Mortola and Maskrey 2011). We performed a pilot study to evaluate the cardiopulmonary response to temporal activation of a G_q_-coupled, M_3_ muscarinic receptor-based DREADD (Designer Receptor Exclusively Activated by Designer Drug) selectively expressed in skeletal muscle (SKM) (Wess et al. 2013). Our study is particularly appropriate for the issue of J Comp Physiol B honouring Dr. Peter Frappell and his insights into metabolic control, cardiopulmonary physiology, as well as many methodologic innovations. Indeed, in 1998 Mortola and Frappell provided a major review of the barometric technique, used in the current study, for measuring pulmonary ventilation, first published by Drorbaugh and Fenn in 1955 for use in human infants, and later extensively used for a range of mammals (Mortola and Frappell 1998).

As an adaptive response to hypoxia, newborns and small adult mammals, including the mouse, respond with a decrease in metabolism, lowering oxygen consumption and dropping their rate of thermogenesis (Frappell et al. 1992; Mortola 2007; Mortola and Maskrey 2011). SKM plays a key role in energy expenditure and metabolism, as well as in maintaining energy balance through the regulation of glucose uptake, disposal and storage (Smith and Muscat 2005; Zurlo et al. 1990). Muscarinic receptors have been implicated in the activation of cellular regulators involved in the adaptive hypoxia response (Hirota et al. 2004) as well as in the ventilatory response to hypoxia (Boudinot et al. 2004). Glucose uptake by SKM is normally mediated through an insulin-dependent mechanism, however, stimulation of skeletal muscarinic M_3_ receptors (M_3_Rs) have been shown to increase glucose uptake in SKM tissue (Merlin et al. 2010). Furthermore, M_3_Rs have been implicated in the regulation of glucose homeostasis, regulation of insulin release and insulin sensitivity (Bone et al. 2019; Gautam et al. 2008; Yamada et al. 2001). Whole-body M_3_R knockout mice (M_3_R^−/−^ mice) fed a high-fat diet display increased oxygen consumption, elevated body temperature and hyperactivity indicative of increased energy expenditure (Gautam et al. 2006).

There is a strong correlation between blood glucose, metabolism and the regulation of body temperature and heart rate (Lo Martire et al. 2018; Mortola and Maskrey 2011; Swoap and Gutilla 2009; Vincent et al. 2007). In many mammals including mice, low blood glucose levels, brought about by an energy imbalance between activity and caloric intake, may play a role in triggering torpor, a state of reduced metabolic demand (Atgie et al. 1990; Lo Martire et al. 2018). This hypometabolic state is accompanied by a reduction in heart rate, respiration, activity and body temperature (Elvert and Heldmaier 2005; Lo Martire et al. 2018; Swoap and Gutilla 2009; Withers 1977). The signaling mechanisms and integrative systems participating in these responses remain areas of significant biologic and potential therapeutic interest. Thus, the presence of a role for skeletal M_3_Rs as a regulatory element in these events remain unclear. Several questions emerge from these observations regarding the in vivo cardiopulmonary and metabolic response to activation of the M_3_R in SKM or the impact of its manipulation through genetic tools altering M_3_R function, which led to the current pilot study.

Recently, Bone and co-workers (Bone et al. 2019) generated a mutant mouse strain that expressed an M_3_R-based DREADD selectively (M_3_Dq) in SKM. The M_3_Dq DREADD does not bind the endogenous ACh transmitter but confers activation through the “designer drug” clozapine N-oxide (CNO), a synthetic ligand which is otherwise pharmacologically inert (Urban and Roth 2015; Wess 2016), thus providing controlled activation of M_3_Dq and G_q_-mediated signaling (Armbruster et al. 2007; Wess et al. 2013). M_3_Dq DREADD mice fed a high-fat diet displayed fasting hyperglycemia, which was dramatically reduced with activation of M_3_Dq receptors by CNO (Bone et al. 2019).

Based on the physiology outlined above and the recent molecular tools to control M_3_R activation, we performed a preliminary study to investigate the impact of the temporal activation of SKM M_3_Dq on in vivo thermoregulation and cardiopulmonary physiology. We addressed the working hypothesis that activation of M_3_Dq DREADD initiates a coordinated cascade of events controlling heart rate, body temperature, and respiratory ventilation that may normally accompany a reduction in metabolism. To test this working hypothesis, we employed DREADD technology in combination with implantable telemetry devices to assess the in vivo responses linked to the SKM M_3_Dq activation.

We addressed two specific hypotheses regarding the M_3_Dq DREADD model:

1. In the absence of CNO, M_3_Dq expression in SKM has no or minimal impact on normal resting cardiopulmonary function.
2. CNO activation of SKM M_3_Dq results in a cardiopulmonary phenotype comprised of a reduction in ventilation, heart rate and core body temperature.

To test these hypotheses, we assessed cardiopulmonary function during activation of SKM M_3_Dq using multiple protocols with DREADD mice. We employed telemetry devices for the measurement of chronic core body temperature (Tb), heart rate (HR) and activity (ACT), whole body plethysmography to evaluate the pattern of breathing in a conscious murine model and finally, an anaesthetized and ventilated mouse model to evaluate the impact of M_3_Dq on respiratory system resistance and compliance.

## Materials and methods

Experimental procedures were approved by Queen’s University Animal Care Committee in accordance with Canadian Council of Animal Care (CCAC) guidelines. On intake all mice were housed in a standard mouse room in the animal care facility, maintained at ~ 22 °C and entrained to a 12 h–12 h light–dark cycle (lights on: 0800 h; lights off 2000 h). Mice were fed a high fat (35.5% fat w/w, Fat calories 60%) diet (F3282, BioServ/Cedarlane Canada) consistent with the diet utilized by Bone et al. (2019). Food and water were supplied ad libitum*.* Mice were housed individually in standard, opaque murine cages (Allentown, Inc., Allentown, NJ, USA) for the duration of the study.

### Animals

The SKM-specific M_3_Dq DREADD mouse model, using the HSA promoter, generated on a C57BL/6NTac background was developed by the National Institute of Diabetes and Digestive and Kidney Diseases (Bone et al. 2019). Tissue expression of M_3_Dq has been previously described [see supplementary data for (Bone et al. 2019)]. M_3_Dq DREADD (*n* = 4, 53.12 ± 1.9 g) and wildtype (WT) control mice (C57BL/6NTac) controls (*n* = 4, 54.1 ± 0.9 g) were supplied through Taconic Farms Inc. (Germantown, NY) and were 36.8 ± 0.1 and 36.7 ± 0.2 weeks old, respectively, at the time of surgical implantation of telemetry device.

### Implantation of telemetry devices

Adult, male M_3_Dq DREADD mice and WT controls were anaesthetized with a mixture of ketamine (80 mg/kg) and xylazine (4 mg/kg) administered intraperitoneally and prepped for sterile aseptic surgery. Mice were placed on a controlled heating pad maintained at 37–39 °C and covered with sterile drapes. Once mice reached a surgical plane of anaesthesia, a small midline abdominal incision was made to allow insertion of the sterile body of an ETA-F10 telemetry device (Data Sciences International (DSI), St. Paul MN, USA) into the abdominal cavity. The ECG leads of the device were secured subcutaneously for optimal ECG recording in a type II configuration at the upper right chest and left side of ribs. The incision was closed using sterile 6-0 polypropylene monofilament sutures. Post-operative fluids and analgesics were given for a period of 3–5 days and mice were allowed to recover for 2 weeks to allow the resumption of normal circadian rhythms and weight gain.

### Circadian variation of physiological variables and impact of DREADD activation

To evaluate the effect of M_3_Dq expression and CNO activation of M_3_Dq on circadian variation of HR, Tb, and ACT, chronically instrumented mice were housed in standard mouse boxes placed on telemetry receiver base (RPC-1 Receiver; Data Science International (DSI), St. Paul MN, USA). Telemetry device signals were collected and stored with a data acquisition system (Dataquest A.R.T, DSI). HR, Tb and ACT values were recorded for 3-min segments at 10-min intervals (Dataquest A.R.T., DSI) and exported to Excel for further analysis. After the re-establishment of normal circadian rhythms, three full 24-h periods were recorded for baseline control measurements. A 24-h average, 10% maximum (representing peak), and 10% minimum (representing trough) measurements were calculated for Tb, HR and ACT for each day then averaged for each individual mouse (Mortola and Seifert 2000; Vincent et al. 2007). Group averages were calculated for each genotype and statistically compared.

Tb, HR and ACT were recorded in a separate protocol for 2 h prior to an intraperitoneal injection of CNO (0.1 mg/kg) and for 22 h post injection. Mice were removed from their cage/receiver; administered a CNO injection and returned within a 5-min period. A two-hour peak response period (100 to 220 min post-CNO injection) was identified and compared to a time-match average 3-day baseline control period of the same duration, for Tb, HR, and ACT.

### Respiratory response to DREADD activation

Implanted mice were injected intraperitoneally with 0.1 mg/kg CNO and placed in a whole-body plethysmograph chamber. Room air flowed through the chamber at a rate of approximately 450 ml/min. The chamber was placed on a telemetry receiver and body temperature (Tb) and heart rate (HR) were recorded continuously via the ETA-F10 telemetry device (Data Science International (DSI), St. Paul MN, USA).

Internal chamber temperature and humidity measurements are made continuously and recorded every 10 min (Vaisala HMP probe, Helsinki, Finland). Pressure changes in the chamber relative to a reference chamber were measured using a differential pressure transducer (Validyne model: MP-45, Validyne Engineering, Northridge, CA, USA) and recorded using a data acquisition system (Spike 2, Cambridge Electronic Design Inc. Cambridge, UK). The plethysmograph chamber was sealed for 90 s every 10 min to allow the measurement of pressure changes due to respiration for a total of 300 to 330 min post injection of CNO. Calibration volumes (0.1 ml) were injected into the sealed chamber at the end of each sampling period. The barometric technique was used to calculate breath-by-breath tidal volume as previously described (Drorbaugh and Fenn 1955; Mortola and Frappell 1998). Other breath-by-breath respiratory variables (*T*_I_, *T*_E_, *T*_tot_, *T*_I_/*T*_tot_, *V*_E_, *V*_T_, *V*_T_/*T*_I_) were calculated using a custom analysis algorithm (Spike 2, Cambridge Electronic Design Inc., Cambridge, UK) and Excel (Microsoft).

We evaluated the impact of CNO activation of M_3_Dq on ventilation and pattern of breathing from breath-by-breath respiratory parameters averaged over a 90 s period recorded every 10 min for 5 h after I.P injection of CNO (0.1 mg/kg).

### Respiratory system mechanics and DREADD activation

A terminal experimental protocol was conducted in anaesthetized, ventilated and paralyzed WT and M_3_Dq DREADD mice to evaluate the effect of M_3_Dq expression and CNO activation of M_3_Dq on respiratory mechanics, respiratory system resistance (Rrs) and compliance (Crs). Mice were anaesthetized with an I.P. injection of sodium pentobarbital (60 mg/kg) and subcutaneous needle electrodes were attached to monitor electrocardiogram (ECG). Body temperature was maintained at 37 °C using a homeothermic blanket system (Harvard Apparatus, USA). Mice were ventilated using a custom small animal ventilator (Model RV5, Voltek Enterprises, Toronto, Canada) set to deliver a 40% oxygen/balance nitrogen gas mixture at a constant inspiratory flow rate of 2.6 ml**/**s. Tidal volume was adjusted at a rate of 7.5 ml**/**kg delivered over 60 ms, with a 185 ms end-inspiratory pause and a 255 ms period of passive expiration (respiratory frequency 120 breaths**/**min). The inclusion of an end-inspiratory pause allows the measurement of airway resistance (Volgyesi et al. 2000). The right jugular vein was cannulated for the intravenous administration of supplemental anaesthetic, paralyzing agent (pancuronium bromide 0.25 mg/kg 0.25 mg/ml), CNO (1.0 mg/kg) and/or methacholine (MCh; 50 µg/kg). ECG, tidal volume, airway flow and airway pressure (*P*_aw_) signals were acquired at a sampling frequency of 2000 Hz using a multi-channel continuous data acquisition and analysis package (Spike 2, Cambridge Electronic Design (CED) Ltd, Cambridge, England). Acquired data were analyzed using CED analysis software and a custom script to generate breath-by-breath measurements of tidal volume, peak *P*_aw_, plateau *P*_aw_, and positive end-expiratory pressure (PEEP). Breath-by-breath maximal respiratory system resistance (Rrs, cmH2O**/**ml s) and compliance (Crs, ml**/**cmH2O) were calculated as reported (Volgyesi et al. 2000). HR was calculated from the R–R interval of the acquired ECG signal.

A 30-s baseline control period was recorded prior to an intravenous injection of CNO 1.0 mg/kg. Rrs and Crs responses (averaged over 240 s) were recorded at 10-min intervals for 1-h post CNO injection. The dosage of CNO utilized was based on previous observations of mortality in mice expressing a Gq-linked designer G-protein coupled receptor selectively in striated muscle (heart and SKM) (Kaiser et al. 2018).

### Data analysis and statistics

The results of each measurement are reported as mean ± SEM. Graphical and numerical analysis of data was performed using Excel (Microsoft, Redmond, WA, USA), SigmaStat 3.0 (Systat Software, San Jose, CA, USA) and GraphPad Prism (V7.03; GraphPad Software, La Jolla, CA, USA). Differences between genotypes for baseline values of Tb, HR and ACT were evaluated using either the Independent *T*-Test (normalized data) or Mann–Whitney *U* test (non-normalized data) following a Shapiro–Wilks Test for normality. Differences between genotypes responses to CNO were evaluated using a two-way ANOVA with Holm-Sidak multiple comparison method post-hoc test. Differences between control values and values post CNO for respiratory parameters, HR, and Tb, were evaluated using One-way Repeated Measures ANOVA with Holm-Sidak multiple comparison method. ANOVA on Ranks was performed where appropriate (Shapiro-Wilks). *p* values of less than 0.05 were considered significant. Post-hoc analysis was performed using Holm-Sidak’s multiple comparison method or Dunn’s multiple comparison method where appropriate.

## Results

### ***M***_***3***_***Dq DREADD expression and circadian rhythm***

**Fig. 1:**
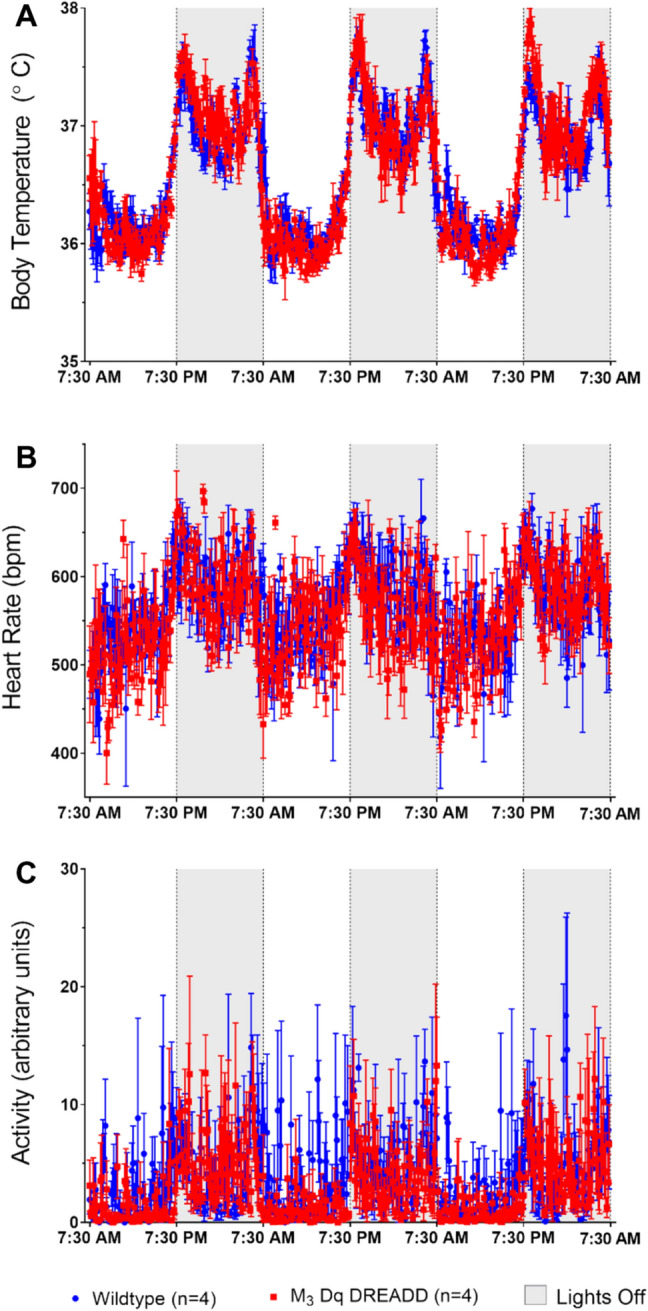
Circadian variation of Tb, HR and ACT. Circadian rhythm of Tb (**A**), HR (**B**) and ACT (**C**) for 3 days in Wildtype mice (blue) and M_3_Dq DREADD mice (red). Both genotypes exhibited a similar entrainment to light and dark (shaded area) cycles. The elevated Tb, HR and ACT during the dark cycle is indicative and expected of a nocturnal species

There was no significant difference in body weight between M_3_Dq DREADD mice and WT mice fed a high-fat diet, consistent with a previous report (Bone et al. 2019). We compared the circadian rhythms of Tb, HR and activity between implanted WT mice and M_3_Dq DREADD mice housed under identical environmental conditions. Both genotypes exhibited similar entrainment to the standard 12–12 h light–dark cycle (Fig. 1).

Both WT and M_3_Dq DREADD mice experienced elevated Tb, HR and ACT during dark periods (lights off), indicative of a wakeful state, and lower Tb, HR and ACT values during the day (lights on), indicative of a resting state which is typical and expected behaviour for a nocturnal species. Body temperature in the M_3_Dq DREADD mice tracked the body temperature of the WT mice closely, including minor variations throughout the day (Fig. 1a). Heart rate (Fig. 1b) and activity (Fig. 1c) were variable, however, over the 3-day period both heart rate and activity in the M_3_Dq DREADD mice trended closely with the WT mice.

A comparison of the 24-h average, 10% maximum and 10% minimum values for Tb and HR between WT and M_3_Dq DREADD mice (Table 1) were not significantly different between genotypes (Independent *T*-Test). The 24-h average and 10% maximum activity levels were not significantly different between the groups.

**Table 1:** Circadian values for control period (prior to CNO activation of M_3_Dq)

Parameter	Wildtype	M3Dq DREADD	*p* value
Body temperature (°C)			
Average	36.59 ± 0.06	36.59 ± 0.06	0.973
10% maximum	37.58 ± 0.05	37.73 ± 0.05	0.200
10% minimum	35.81 ± 0.08	35.73 ± 0.05	0.402
Heart Rate (bpm)			
Average	564.8 ± 10.3	555.6 ± 7.1	0.492
10% maximum	677.7 ± 12.1	685.2 ± 5.2	0.592
10% minimum	438.8 ± 13.4	427.7 ± 11.4	0.550
Activity (arbitrary units)			
Average	4.0 ± 0.6	3.0 ± 0.5	0.263
10% maximum	17.1 ± 1.8	13.9 ± 2.0	0.285

Values for 24-h average, 10% minimum, and 10% maximum are reported for Tb, HR and ACT. There was no significant difference between genotypes for any value (Independent *T*-test)

### ***Impact of M***_***3***_***Dq activation by CNO on circadian variables***

**Fig. 2:**
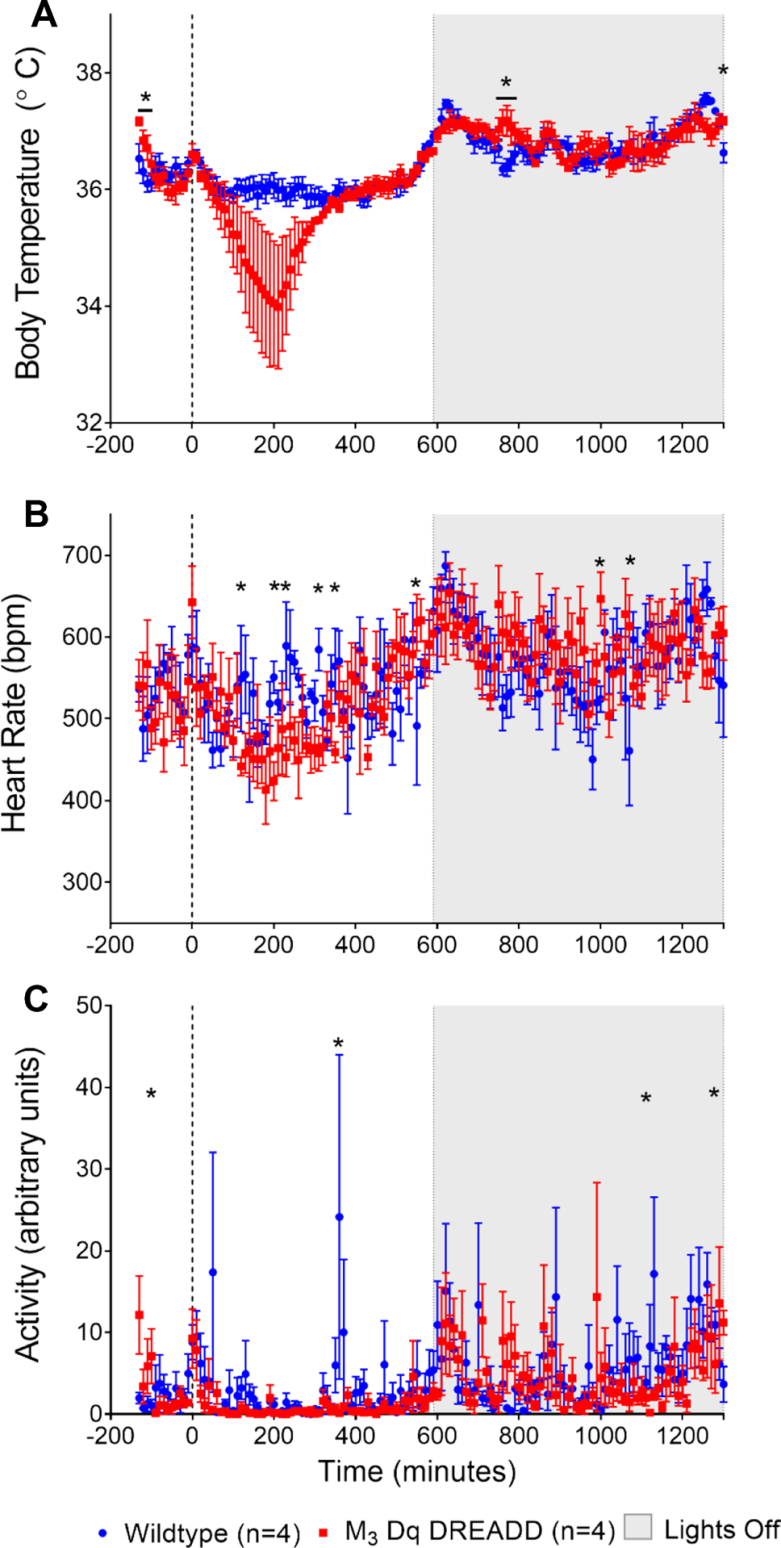
Average 24 h Tb response to CNO. Average 24 h Tb (**A**), HR (**B**) and ACT (**C**) response to CNO (0.1 mg/kg, Time 0) in wildtype mice (blue) and M_3_Dq DREADD mice (red). CNO activation of M_3_Dq elicited a variable but profound decrease in Tb and a reduction in HR and ACT compared to the wildtype. Average ± SEM (Two-way ANOVA on ranked data, Holm-Sidak multiple comparison; asterisks indicate a significant difference between genotypes *p* < 0.05)

The response to activation of the M_3_Dq via 0.1 mg/kg CNO was recorded for 24 h post intraperitoneal injection. Figure 2 illustrates the average Tb (a), HR (b) and ACT (c) values, taken every 10 min from 2 h prior to CNO injection to 24 h post injection, in the WT and M_3_Dq DREADD mice.

CNO had no effect on WT mice, which exhibited normal circadian variability in Tb over the 24-h period. In comparison to WT mice, the magnitude of the Tb response to M_3_Dq activation by CNO, in the M_3_Dq DREADD mice was variable, but profound (Fig. 2a). The response initiated approximately 60 min after CNO injection and lasted for approximately 5 h (300 min). The average Tb nadir in the M_3_Dq DREADD was 34 °C (range 31.5–35.4 °C). Of the four M_3_Dq DREADD mice, two responded strongly, with a minimum Tb of 32.7 and 31.5 °C. The remaining two low-responders (0.5 and 0.6 °C drop in Tb) responded to a separate injection of 0.3 mg/kg CNO more robustly with a greater drop in Tb (0.7–1.5 °C drop in Tb; data not shown) consistent with the observation that activation of M_3_Dq DREADD varies with CNO dose as previously reported (Kaiser et al. 2018). There was no significant overall difference in the 24-h mean Tb values among genotypes (Two-way ANOVA on ranked data, Holm-Sidak multiple comparison; *p* = 0.101). Post-hoc analysis showed specific periods (− 130 to − 110 and 760–780 and 1300 min post injection) were significantly different between genotypes (Fig. 2a, significance indicated by asterisks), however, these periods were prior to injection and more than 10 h after CNO injection, therefore the physiologic significance of these differences remains unclear. Approximately 6 h (340 min) after activation of M_3_Dq by CNO, the Tb in the M_3_Dq DREADD mice returned to trend closely to the Tb values seen in the WT mice.

The HR response to activation of the M_3_Dq in the M_3_Dq DREADD mice resulted in an over-all drop in HR of approximately 50–100 bpm from WT values (Fig. 2b). Time points 120, 200, 230, 310, 350 min post injection were significantly different between genotypes (*p* = 0.04, 0.016, 0.01, 0.016 and 0.046, respectively). The variability in HR resulted in no significant difference in the mean 24-h HR values between WT and M_3_Dq DREADD (Two-way ANOVA, Holm-Sidak multiple comparisons; *p* = 0.476) (Fig. 2b, asterisks indicate individual significant time points).

A sharp spike in activity levels in both M_3_Dq DREADD and WT mice just after time 0 likely reflects the increased activity due to restraint and injection (Fig. 2c). In general, activity levels were highly variable in both genotypes. However, activity levels in the M_3_Dq mice were lower than WT mice in the hours after CNO injection. The mean 24 h ACT response to activation of the M_3_Dq in the M_3_Dq DREADD mice resulted in a significantly lower mean ACT level compared to mean ACT levels in the WT mice (*p* < 0.001; Two-way ANOVA on ranked data, Holm-Sidak multiple comparisons).

**Fig. 3:**
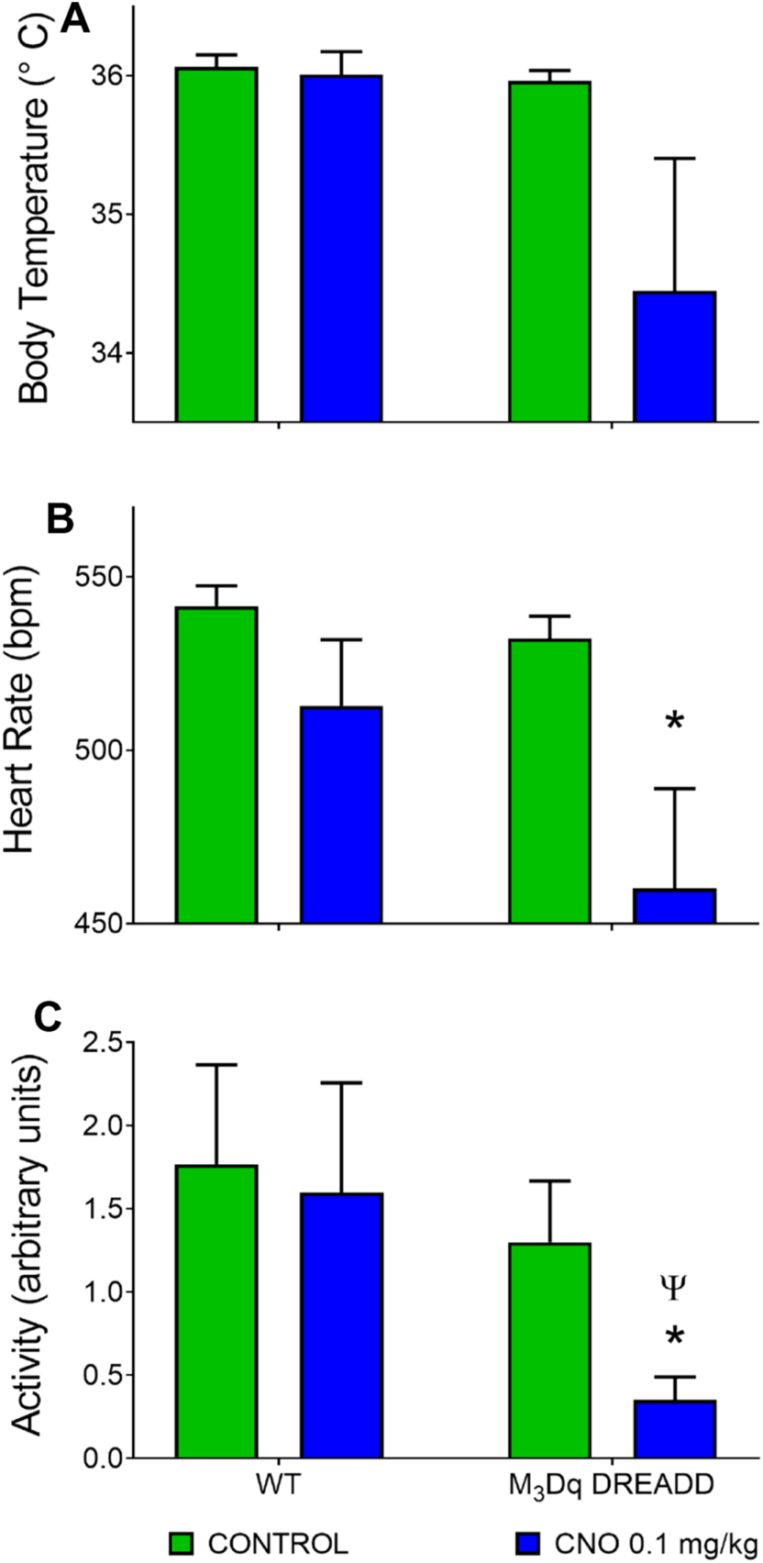
Peak Tb, HR and ACT response to CNO compared to control. Tb (**A**), HR (**B**) and ACT (**C**) response to maximal activation of M_3_Dq (110–220 min post CNO; blue bars) in WT and M_3_Dq DREADD mice compared to a time-matched control period (green bars). * = significant difference between control and max response, *p* < 0.05. Ψ = significant difference between genotypes, *p* < 0.05 (Two-way ANOVA on ranks, Holm-Sidak multiple comparison)

Taking into consideration the pharmacokinetics of CNO and after examining the timing of the nadir responses, we arbitrarily selected a 120-min period from 100 to 220 min post CNO injection in both WT and M_3_Dq DREADD mice to represent the “peak” or period of maximal activation of M_3_Dq. A 120-min time of day-match baseline control period was obtained for each individual mouse from the 3-day average baseline control recorded previously. Average peak Tb (a), HR (b) and ACT (c) values were compared with average controls (Fig. 3). There was no significant difference in baseline-control values between M_3_Dq DREADD and WT mice (green bars; Fig. 3) for Tb (*p* = 0.288), HR (*p* = 0.269) or ACT values (*p* = 0.524). There was also no significant difference between the peak response and control values in the WT mice for Tb (*p* = 0.2588), HR (*p* = 0.091) or ACT (*p* = 0.524; Two-way ANOVA on ranks, Holm-Sidak multiple comparisons), illustrating the ineffectiveness of CNO in the WT mouse.

Activation of M_3_Dq by CNO in the M_3_Dq DREADD mice produced a significant drop in heart rate (*p* = 0.033; Fig. 3b) and activity (*p* = 0.026; Fig. 3c) compared to control values (asterisk denotes significance). Activity level in the M_3_Dq DREADD mice in response to activation of M_3_Dq was significantly lower compared to the activity level seen in the WT mice (*p* = 0.026; Fig. 3c, Psi denotes significance). Tb decreased compared to control values in response to activation of M_3_Dq but was not significantly different (*p* = 0.258) at a dose of 0.1 mg/kg CNO (Fig. 3a). In two of the M_3_Dq DREADD mice tested, the 0.1 mg/kg caused a robust response, whereas in two M_3_Dq DREADD mice a dose of 0.3 mg/kg was required to produce a threshold response. This may reflect biological variations in DREADD threshold responses or other factors, but note that analysis of the Tb data based on threshold response would change the statistical outcome (max response 33.93 ± 0.70 °C vs. control 35.96 ± 0.07 °C; *p* = 0.003; Two-way ANOVA with Holm-Sidak multiple comparisons).

### ***Impact of M***_***3***_***Dq activation by CNO on the pattern of breathing***

M_3_Dq DREADD mice were injected with CNO immediately prior to placement in the whole-body plethysmograph chamber. Based on the 60-min delayed onset of CNO activation observed with the circadian rhythm protocol, we chose to average the first five samples (50 min) as representative of control values (CON) for each parameter.

**Fig. 4:**
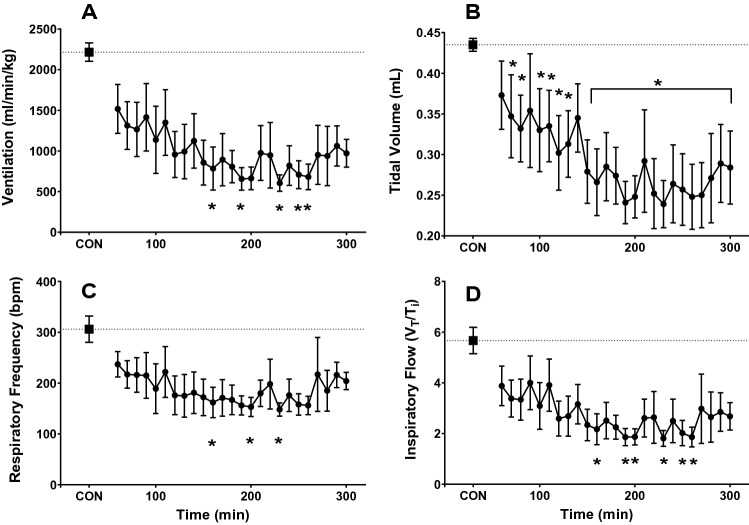
Pattern of breathing response to activation of M_3_Dq by CNO. Average ventilation (**A**), tidal volume (**B**), respiratory frequency (**C**), and inspiratory flow (**D**) response to activation of M_3_Dq by CNO (0.1 mg/kg). Mice were injected with CNO immediately prior to placement in the whole-body plethysmograph. Samples were recorded every 10 min for 5 h. The average of the first 50 min represents the control values (CON). * = significantly different from control values (*p* < 0.05; one-way RM ANOVA)

Ventilation (*V*_E_) decreased rapidly from control values (Fig. 4a; asterisks denote significance from control) upon activation of skeletal M_3_Dq by CNO. Median values were significantly lower than control (*p* = 0.036 One-way RM ANOVA on ranks, Dunn’s multiple comparisons). The ventilatory response to M_3_Dq activation by CNO was reflected in a significant depression in both tidal volume (*V*_T_; Fig. 4b) and respiratory frequency (*f*; Fig. 4c). Mean *V*_T_ (*p* ≤ 0.001) and *f* (*p* = 0.032) values were significantly lower compared to control values (One-way RM ANOVA, Holm-Sidak and Dunn’s multiple comparisons, respectively).

Median values for inspiratory flow (*V*_T_/*T*_I_; Fig. 4d, *p* = 0.002) were significantly lower compared to control values (One-way RM ANOVA on ranks, Dunn’s multiple comparisons).

**Fig. 5:**
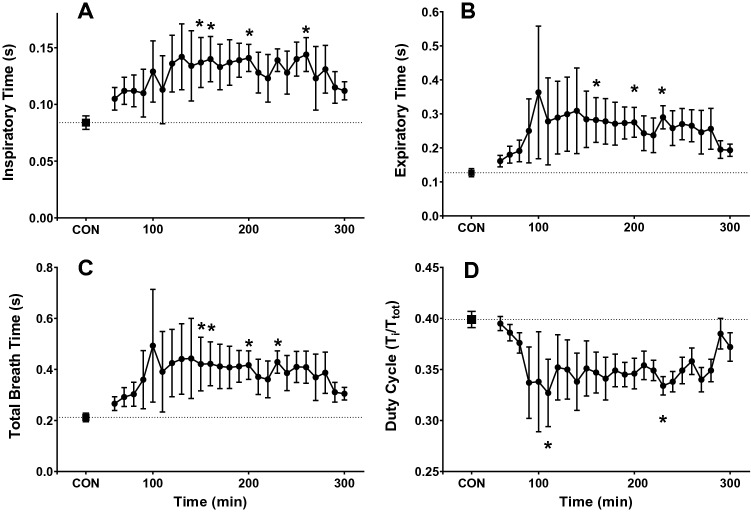
Respiratory timing response to activation of M_3_Dq by CNO. Average inspiratory time (**A**), expiratory time (**B**), total breath time (**C**), and duty cycle (**D**) response to CNO (0.1 mg/kg) in M_3_Dq DREADD mice. Mice were injected with CNO immediately prior to placement in the whole-body plethysmograph. Samples were recorded every 10 min for 5 h. The average of the first 50 min represents the control values (CON). * = significantly different from control values (*p* < 0.05; One-way RM ANOVA)

Both inspiratory (*T*_I_; Fig. 5a) and expiratory time (*T*_E_; Fig. 5b) increased compared to control, as reflected in a longer total breath time (*T*_tot_; Fig. 5c) and a shorter duty cycle (*T*_I_/*T*_tot_; Fig. 5d). Median values for *T*_I_ (*p* = 0.01), *T*_E_ (*p* = 0.03), *T*_tot_ (*p* = 0.027) and *T*_I_/*T*_tot_ (*p* = 0.042) were significantly different from control values (One-way RM ANOVA on ranks, Dunn’s multiple comparisons); specific time point differences are indicated by an asterisk, (*p* < 0.05).

The nadir for ventilation was reached at 230 min post-CNO injection and ventilation remained depressed compared to control values at 5 h. The effects of activation of M_3_Dq by CNO on ventilation appear to be driven primarily by changes in tidal volume as the effect on respiratory frequency and associated values of *T*_TOT_, *T*_I_, *T*_E_ and *T*_I_/*T*_tot_ appear transient and begin to approach control values after 5 h.

Like our findings for the circadian protocol, HR and Tb decreased from control values (data not shown). Median values during the 5-h recording were significantly lower than control values for both HR (*p* ≤ 0.001) and body Tb (*p* ≤ 0.001; One-way RM ANOVA on ranks, Dunn’s multiple comparison method).

### ***Impact of M***_***3***_***Dq activation by CNO on respiratory resistance and compliance***

Mean baseline control values (Pre-CNO) for respiratory system resistance (Rrs) were not significantly different between WT and M_3_Dq DREADD mice (0.974 ± 0.032 vs 1.156 ± 0.089, respectively; *p* = 0.103 Independent *T*-Test). Similarly, mean baseline control values for compliance (Crs) were not significantly different between WT and M_3_Dq DREADD mice (0.030 ± 0.003 vs 0.028 ± 0.002, respectively; *p* = 0.651; Independent *T*-test**).**

**Fig. 6:**
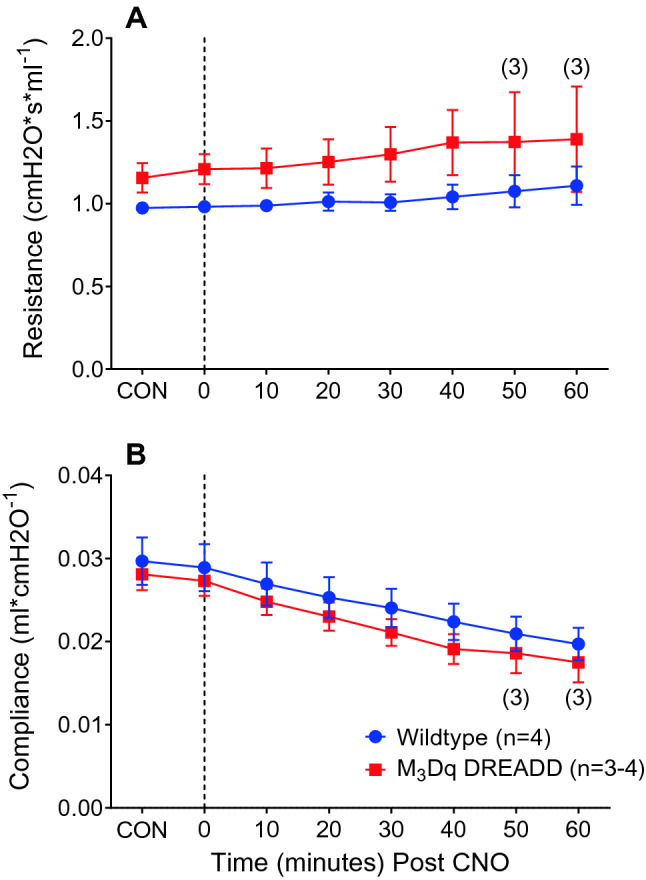
Average resistance and compliance response to activation of M_3_Dq by CNO. Average airway resistance (**A**) and compliance (**B**) response to activation of M_3_Dq by CNO (1.0 mg/kg, i.v. at time 0) in wildtype (blue circle) and M_3_Dq DREADD mice (red square). Numbers in brackets represent a number of mice in average

Figure 6 illustrates the average Rrs and Crs values recorded every 10 min from 10 min before, until 60 min after, 1.0 mg/kg CNO injected intravenously. In the WT mice, Rrs remained relatively stable with little in-group variability for the 60-min following CNO administration (Fig. 6a). The Rrs response in the M_3_Dq DREADD mice was variable within the group, with two mice displaying less than a 10% change in Rrs compared to control, and another displaying a 56% increase in Rrs at time 60 min. A fourth mouse succumbed to cardiac arrest at 47 min post CNO. On average, 60 min after activation of M_3_Dq by CNO, Rrs increased to a peak of 19% from control values in the M_3_Dq DREADD mice, compared to a 13% increase in Rrs values in the WT mice. A Two-way ANOVA on ranks (Holm-Sidak post hoc) statistical analysis was significant for the overall mean Rrs (*p* = 0.006) between genotypes, however, there was no statistical difference between any specific time points.

Both WT and M_3_Dq DREADD mice displayed a time-dependent decrease in lung compliance consistent with that expected from a constant volume history (Mead and Collier 1959) (Fig. 6b). On average, 60 min after activation of M_3_Dq by CNO, Crs decreased by 35% from control in the M_3_Dq DREADD mice, compared to a 33% decrease from control in the WT mice. There was no statistically significant difference between mean Crs response between genotypes (*p* = 0.078; Two-way ANOVA on ranks, Holm-Sidak post hoc).

### ***Electrocardiogram observations on M***_***3***_***Dq activation by CNO***

**Fig. 7:**
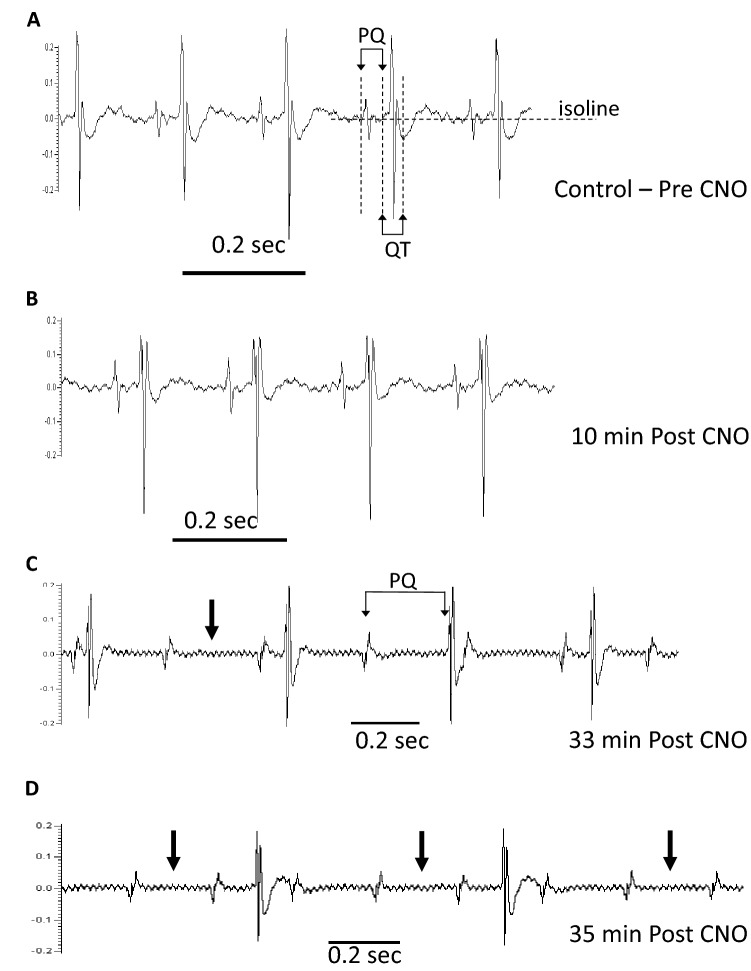
Electrocardiogram observations on M_3_Dq activation by CNO. ECG recording from an anesthetized M_3_Dq DREADD mouse before (**A**), 10 min (**B**), 33 min (**C**), and 35 min (**D**) after intravenous injection of 1 mg/kg CNO. ECG disorders include irregular and elongated PQ intervals as well as the absence of QRS complexes (arrows; **C** and **D**)

CNO-induced dose-dependent mortality has been reported in mice expressing M_3_Dq in both heart and SKM tissue (Kaiser et al. 2018). We examined the previously recorded ECG pattern of the M_3_Dq DREADD mouse that succumbed to cardiac arrest. In response to a CNO dose of 0.1 mg/kg, an irregular HR was noted in the conscious recording approximately 110 min post injection with normal HR rhythm resuming approximately 5 h after CNO injection. At a higher dose of CNO (1.0 mg/kg), the anaesthetized mouse developed a fatal arrhythmia approximately 47 min post injection. The PQ and QT intervals, measured as previously reported (Zhang et al. 2014), were evaluated in the anaesthetized recording, immediately before (a), and 10 (b), 33 (c) and 35 min (d) post-CNO injection (Fig. 7). QT intervals lengthened, compared to pre-CNO values, at approximately 35 min post CNO. Irregular and prolonged PQ intervals (Fig. 7c) indicative of atrioventricular (AV) second-degree block type 1 occurred at approximately 33 min post-CNO injection. A ten-fold difference in PQ intervals was noted between pre-CNO and 33 min post CNO. Arrhythmias progressed to AV second degree block type 2, with the intermittent absence of QRS complexes (Fig. 7c, d), tachycardia and eventually the death of the animal at 47 min (data not shown).

These observations illustrate the in vivo pattern of ECG changes responsible for lethal heart block reported with high dose CNO exposures consistent with the observations made by Kaiser et al. (2018).

## Discussion

In the present study, we demonstrated that activation of SKM M_3_Dq by CNO in M_3_Dq DREADD mice, resulted in a coordinated physiologic response comprised of a reduction in Tb, HR, ACT, and pulmonary ventilation, which is consistent with a reduction in metabolism. Skeletal muscle plays a major role in metabolism and energy expenditure (Zurlo et al. 1990) and a recent study demonstrating the impact of SKM M_3_Dq activation by CNO on diet-induced hyperglycemia and glucose homeostasis (Bone et al. 2019) has raised the question of the potential role of skeletal M_3_Rs in the generation of hypometabolic events associated with the initiation of torpor (Lo Martire et al. 2018). Furthermore, studies using a Gq-coupled DREADD (M_3_Dq) in striated muscle (heart and SKM), have raised questions regarding the impact on cardiopulmonary physiology of the activation of skeletal M_3_Dq (Kaiser et al. 2018). Our study used the Gq DREADD model to activate, in a controlled manner, the signaling pathway of Gq-coupled receptors expressed in skeletal muscle and it would be reasonable to hypothesize that activation of any Gq-coupled receptors expressed in skeletal muscle could impact cardiopulmonary health and metabolic responses in the manner reported herein. It remains to be seen whether the nature of pathologies associated with activation of the Gq signaling may be linked to thermoregulation or cardiopulmonary changes coupled to M_3_R or other regulation of SKM function. However, the physiological mechanisms initiated by selective Gq DREADD activation are clearly functional and profound in their effects. Further, the DREADD model provides a unique and powerful tool to explore novel roles of skeletal M_3_ and other receptor functions in cardiopulmonary health and disease.

Bone et al. previously reported no differences between M_3_Dq DREADD mice and WT mice in terms of body weight, glycemia or plasma insulin levels (Bone et al. 2019). We extend these observations to show that expression of skeletal M_3_Dq DREADD has no impact on normal circadian variation of Tb, HR or ACT, resting minute ventilation or on baseline respiratory mechanics (Rrs and Crs). Based on multiple protocols, we report for the first time, the pattern of changes associated with CNO activation of skeletal M_3_Dq. Hypothermia, bradycardia, reduced activity, and a reduction in pulmonary ventilation are responses suggestive of a reduction in metabolism similar to the responses seen in torpor or the adaptive response to hypoxia in mice (Elvert and Heldmaier 2005; Frappell et al. 1992; Swoap 2008). We speculate based on reports of others (Bone et al. 2019) that activation of skeletal M_3_Dq receptors brought about an increase in glucose uptake to SKM tissue, triggering a hypoglycemia-induced reduction in metabolism. Indeed, the hypoglycemic consequence seen with high-level activation of skeletal M_3_Dq may be responsible for the detrimental cardiac arrhythmias (Kaiser et al. 2018). Further studies are required to explore the hypotheses emerging from our pilot studies.

In humans, there is some evidence linking hypothermia to hypoglycemic events in patients with diabetes (Naseerullah and Murthy 2018; Tran et al. 2012). In many small mammals, hypoglycemia and the fall of blood glucose levels are associated with the initiation of spontaneous torpor or hibernation and a hypometabolic state characterized by a reduction in body temperature and activity (Atgie et al. 1990; Dark et al. 1999; Lo Martire et al. 2018). Similarly, a hypometabolic state with the associated reduction of body temperature is an adaptive response to hypoxia seen in small-bodied mammals such as mice (Frappell et al. 1992). Activation of skeletal M_3_Dq-mediated Gq signaling has been shown to promote SKM glucose uptake (Bone et al. 2019; Merlin et al. 2010). Bone et al. (2019) reported a dramatic reduction in fasting blood glucose levels with CNO activation of skeletal M_3_Dq related to glucose uptake by SKM.

Hypoxia has been shown to reduce blood glucose levels independent of diminished weight gain and although the exact mechanism is unknown, it has been speculated that growth factors may play a causal role (Abu Eid et al. 2018). Muscarinic receptors have been implicated in the activation of cellular regulators involved in the adaptive hypoxia response (Hirota et al. 2004) suggestive of a link between muscarinic receptors and metabolic control. Though not specifically measured in this study, our data support the notion that activation of skeletal M_3_Dq lowers blood glucose levels sufficiently to elicit a hypometabolic state.

In this study, activation of skeletal M_3_Dq by CNO triggered bradycardia and in one mouse a fatal cardiac arrhythmia. Indeed cardiac arrhythmia and mortality have been reported with the activation of striated muscle M_3_Rs in a DREADD model (Kaiser et al. 2018). Cardiac arrhythmia has been linked to hypoglycemia in both humans and other species (Hanefeld et al. 2016; Kacheva et al. 2017; Reno et al. 2017; Robinson et al. 2003). In the hypometabolic state of torpor, a reduction in heart rate accompanies the reduction in body temperature and metabolism (Elvert and Heldmaier 2005; Swoap and Gutilla 2009). Our observation in which prolongation of the PQ interval was observed in a single animal, is reminiscent of that reported for dormice entering torpor, in which they develop cardiac arrhythmia characterized by extra systoles and the prolongation of the PT duration (Elvert and Heldmaier 2005).

## Conclusion

Our pilot study embraces the themes championed by Professor Peter Frappell coupled with his talented students and collaborators, who explored hypothesis-driven experimentation for mammalian cardiorespiratory biology and the use of chronic measurements across a broad range of species. We conclude that selective activation of skeletal M_3_Dq by CNO elicits a constellation of physiologic responses that reduce pulmonary ventilation, body temperature, heart rate, and activity in a coordinated manner consistent with a decrease in metabolism. Further experiments are required to determine whether M_3_Dq-initiated pathways play a role in the link between skeletal muscle modulation of glucose homeostasis and metabolism seen in either acute hypoxia or torpor, which are both accompanied by a similar cardiopulmonary phenotype.

## Data Availability

The datasets generated during and/or analyzed during the current study are available from the corresponding author on reasonable request.
